# The role of sleep and wakefulness in myelin plasticity

**DOI:** 10.1002/glia.23667

**Published:** 2019-06-25

**Authors:** Luisa de Vivo, Michele Bellesi

**Affiliations:** ^1^ School of Physiology, Pharmacology and Neuroscience University of Bristol Bristol UK

**Keywords:** brain, myelin, oligodendrocyte, sleep deprivation, white matter

## Abstract

Myelin plasticity is gaining increasing recognition as an essential partner to synaptic plasticity, which mediates experience‐dependent brain structure and function. However, how neural activity induces adaptive myelination and which mechanisms are involved remain open questions. More than two decades of transcriptomic studies in rodents have revealed that hundreds of brain transcripts change their expression in relation to the sleep–wake cycle. These studies consistently report upregulation of myelin‐related genes during sleep, suggesting that sleep represents a window of opportunity during which myelination occurs. In this review, we summarize recent molecular and morphological studies detailing the dependence of myelin dynamics after sleep, wake, and chronic sleep loss, a condition that can affect myelin substantially. We present novel data about the effects of sleep loss on the node of Ranvier length and provide a hypothetical mechanism through which myelin changes in response to sleep loss. Finally, we discuss the current findings in humans, which appear to confirm the important role of sleep in promoting white matter integrity.

## 1 INTRODUCTION

Oligodendrocytes are the central nervous system (CNS) cells that produce myelin, an electrically insulating substance that enwraps axons and allows fast propagation of nerve pulses (Armati and Mathey, 2010). Oligodendrocytes derive from oligodendrocyte precursor cells (OPCs), which represent the largest pool of immature cells and the major source of newly formed mature oligodendrocytes in the CNS (Richardson et al., 2011; Dimou and Gallo, 2015). These cells abundantly proliferate during development, migrating from the neural tube in what it will become the white matter of the CNS. During the migration process, OPCs continuously expand and retract their processes in the attempt to sense neighbor OPCs so that the cells can distribute uniformly throughout the tissue (Simons and Trotter, 2007). OPCs continue to proliferate also in the adult CNS, although their proliferation rate declines as the animal ages (Psachoulia et al., 2009).

The classical organization of myelin consists of multiple myelin segments along the axon, separated by regions devoid of myelin called nodes of Ranvier. However, the proportion of the axon that is myelinated can vary in the brain. A recent study investigated myelination in the mouse cerebral cortex using three‐dimensional electron microscopy and found that the classical distinction in myelinated and unmyelinated axons is outdated (Tomassy et al., 2014). In fact, axons can show myelinated segments intermittently separated by long unmyelinated segments (Tomassy et al., 2014), indicating that myelination is a phenomenon more complex than previously thought, at least in the cerebral cortex.

The way oligodendrocytes myelinate the axon has been recently elucidated in a study using an integrative approach of live imaging, electron microscopy, and genetics in zebrafish (Snaidero et al., 2014). Deposition of new myelin layers occurs by continuous spiralling of the innermost tongue of the myelin around the axon. Simultaneously, these layers extend laterally along the axonal length, finally leading to the formation of a group of closely apposed paranodal loops (Snaidero et al., 2014). Unlike Schwan cells (the myelinating cells of the peripheral nervous system), a single oligodendrocyte can enwrap multiple axons. It has been estimated that an individual oligodendrocyte can maintain up to 60 internodal segments (Remahl and Hilderbrand, 1990), thus sustaining a high metabolic cost to support such large surface area of plasma membrane. By preventing current leakage and reducing membrane capacitance, myelinated segments facilitate rapid saltatory conduction along the axon at speeds 50‐ to 100‐fold faster than unmyelinated axons of the same calibre (Zalc and Colman, 2000; Zalc et al., 2008). Fast propagation allows for precise spike‐time arrival thus contributing to appropriate neuronal signalling integration required to coordinate sensorimotor and cognitive functions (Fields, 2015).

In any given axon, four key factors determine the speed at which an action potential travels along the axon (conduction velocity) (Chorghay et al., 2018): (1) the axon diameter; (2) the myelin thickness, often measured by g‐ratio (e.g., the ratio of the inner to the outer diameter of the fibre) (Rushton, 1951; Waxman, 1975); (3) the length of continuous axonal segment that is myelinated (i.e., the internode distance or length) (Rushton, 1951; Brill et al., 1977); (4) the nodal geometry itself (Arancibia‐Cárcamo et al., 2017). By regulating these factors, conduction velocity can be adjusted, an advantageous mechanism that increases the functional adaptation of the CNS to environmental experience (Forbes and Gallo, 2017).

The traditional view that considers myelin as being static and modifiable only by damage is no longer valid. In recent years, a growing body of evidence has shown that myelin and the cells that form it, from OPCs to mature myelinating oligodendrocytes, are remarkably dynamic and responsive to the neurons they ensheathe (Aggarwal et al., 2011; Fields, 2015; Forbes and Gallo, 2017; Kaller et al., 2017; Mount and Monje, 2017; Monje, 2018). Artificially induced neuronal activity using selective optogenetic stimulation in premotor cortex generated a rapid and robust proliferation of OPCs followed by increased myelination in the deep cortex and subcortical white matter within the stimulated circuit (Gibson et al., 2014). By contrast, reducing sensory input by whisker‐trimming at birth lead to diminished myelination a few weeks later in the barrel cortex of the contralateral hemisphere (Barrera et al., 2013). Similarly, decreased social experience in juvenile mice resulted in remarkably reduced myelin in the prefrontal cortex. Interestingly, this deficit could not be rescued by stopping social isolation later on in late adolescence, indicating the existence of a critical period of myelin vulnerability during development (Liu et al., 2012).

Learning can induce myelin changes. For example, rats trained in a single‐pellet reaching task exhibit changes in MRI correlates of white matter microstructure modification, and augmented histological expression of myelin basic protein in the cingulum and external capsule, two regions characterized by numerous fibers projecting to brain regions strategically important for motor learning (Sampaio‐Baptista et al., 2013). Further support for the link between myelin changes and learning comes from a recent study showing that mice lacking the ability to generate new myelinating oligodendrocytes failed to learn a complex motor task, thus indicating that new myelin deposition is a necessary requirement for the acquisition of new motor skills (McKenzie et al., 2014; Xiao et al., 2016). In humans, learning a new task, such as juggling, induced white matter changes in areas involved in visuomotor integration (Scholz et al., 2009). Moreover, significant changes have been also observed in left white matter tracts of anterior hemisphere of old adults before and after 8 weeks of memory training (Engvig et al., 2012), thus suggesting that white matter is modifiable not only after motor learning.

Thus, the dynamic responses of myelin‐forming cells to neuronal activity, sensory input, and learning constitute a critical type of brain plasticity, that is, myelin plasticity, which contributes to the optimization of neuronal circuitry, helps consolidate motor and cognitive function, and permits the acquisition of new skills. How the environment, experience, and activity influence myelination and what factors regulate myelin plasticity are still open questions.

## 2 SLEEP AND MYELIN

### 2.1 Evidence from gene expression studies

Sleep is a highly conserved behavior, universally present throughout the animal kingdom and occupying a substantial proportion of each animal's lifetime (Tobler, 2011). This indicates that sleep must serve some fundamental functions for the organism, some of which remain to be fully elucidated (Cirelli and Tononi, 2008). Much of what we have learnt about sleep's cellular consequences comes from two decades of gene expression studies, which highlighted differences in gene transcription between the two major behavioral states: sleep and wake (Cirelli and Tononi, 2000; Cirelli et al., 2004, 2005, 2006; Terao et al., 2006; Maret et al., 2007; Mongrain et al., 2010; Vecsey et al., 2012; O'Callaghan et al., 2018). Interestingly, these studies consistently highlight that genes implicated in the maintenance of cellular plasma membranes are preferability transcribed during sleep suggesting that, perhaps, one of the functions of sleep could be to maintain healthy plasma membranes. Given the vast amount of plasma membrane that oligodendrocytes have to sustain, it is not surprising that many of these plasma membrane‐related genes were specific for myelin.

In 2004, Cirelli and collaborators found that several genes coding for myelin structural proteins (MOBP, MAG, plasmolipin, CD9), myelin‐related receptors (insulin‐like growth factor binding protein 2), and enzymes (2′:3′‐cyclic nucleotide‐3′‐phosphodiesterase, Na/K ATPase subunit α2, methionine adenosyltransferase, carbonic anhydrase II) were specifically upregulated over the course of several hours of sleep. Likewise, sleep upregulated the transcription of genes coding for enzymes involved in the synthesis and transport of cholesterol, a major constituent of myelin and other membranes (thiolase, 3‐hydroxy‐3‐methylglutaryl‐Coenzyme A synthase, squalene synthase, lanosterol 14 α‐demethylase) (Cirelli et al., 2004). A few years later, another study found a similar association between sleep and myelin‐related genes. Importantly, in this study adrenalectomized mice were used as an additional control group to reduce possible confounding effects of corticosterone‐related stress associated with the sleep deprivation procedure (Mongrain et al., 2010). Pair‐wise comparisons between adrenalectomized sleep deprived and sleeping mice confirmed the downregulation of some myelin‐related transcripts (e.g., *opalin* and *plasmolipin*), indicating that their reduced expression was rather due to the lack of sleep than corticosterone‐related stress (Mongrain et al., 2010). Furthermore, *plasmolipin* in association with CD9, a gene coding for a membrane protein normally expressed in the mature myelin sheath, was also found downregulated in cortical samples of rats subjected to sleep deprivation for seven days (Cirelli et al., 2006), again suggesting that also chronic lack of sleep inhibits the transcription of proteins involved in myelination.

More recently, using a novel transcriptomic technique (translating ribosome affinity purification, TRAP) in conjunction with microarray analysis, we carried out a genome‐wide messenger RNA profiling of the oligodendrocyte lineage as a function of sleep, wake, and short sleep‐deprivation in mouse forebrain samples (Bellesi et al., 2013). Unlike previous gene expression studies, this method allowed us to evaluate the transcriptomic changes specifically in oligodendrocytes. In addition, since the TRAP method targets mRNAs already attached to ribosomes, this technique enabled us to obtain the entire translated mRNA of oligodendrocytes, providing a better understanding of the cellular functions that are modulated by sleep and wake. The gene expression analysis revealed that more than 2% of all genes expressed in oligodendrocytes changed their expression because of the sleep/wake cycle, independent of time of day. Specifically, we identified 310 sleep genes (0.9%) and 404 wake genes (1.2%), which belonged to different functional categories.

Clustering analysis confirmed that many genes, overexpressed during sleep, were indeed involved in plasma membrane maintenance (Bellesi et al., 2013). Among these, many were involved specifically in lipid metabolism, in particular in glycerophospholipid biosynthesis, such as *Dgat2*, *Cds1*, *Elovl7*, *Chka*, while others in myelination (e.g., *Opalin*, *Plasmolipin*, *Qk*). Of note, both *Opalin* and *Plasmolipin* were found upregulated in sleep also by other studies (Cirelli et al., 2006; Mongrain et al., 2010). Opalin is thought to help myelin stabilization at the paranodal region (Yoshikawa et al., 2008), while Plasmolipin participates in the biogenesis of myelin by forming membrane domains at the Golgi complex thanks to its capacity of attracting cholesterol and sphingolipids (Yaffe et al., 2015). Finally, *Qk* codes for an RNA‐binding protein that posttranscriptionally regulates mRNA stability and distribution of several myelin‐associated transcripts, including *Mbp* and *Mag* (Zearfoss et al., 2008). Consequently, mice lacking Qk (quaking viable mice) display a remarkable deficit of compact myelin (Zearfoss et al., 2008).

**Figure 1:**
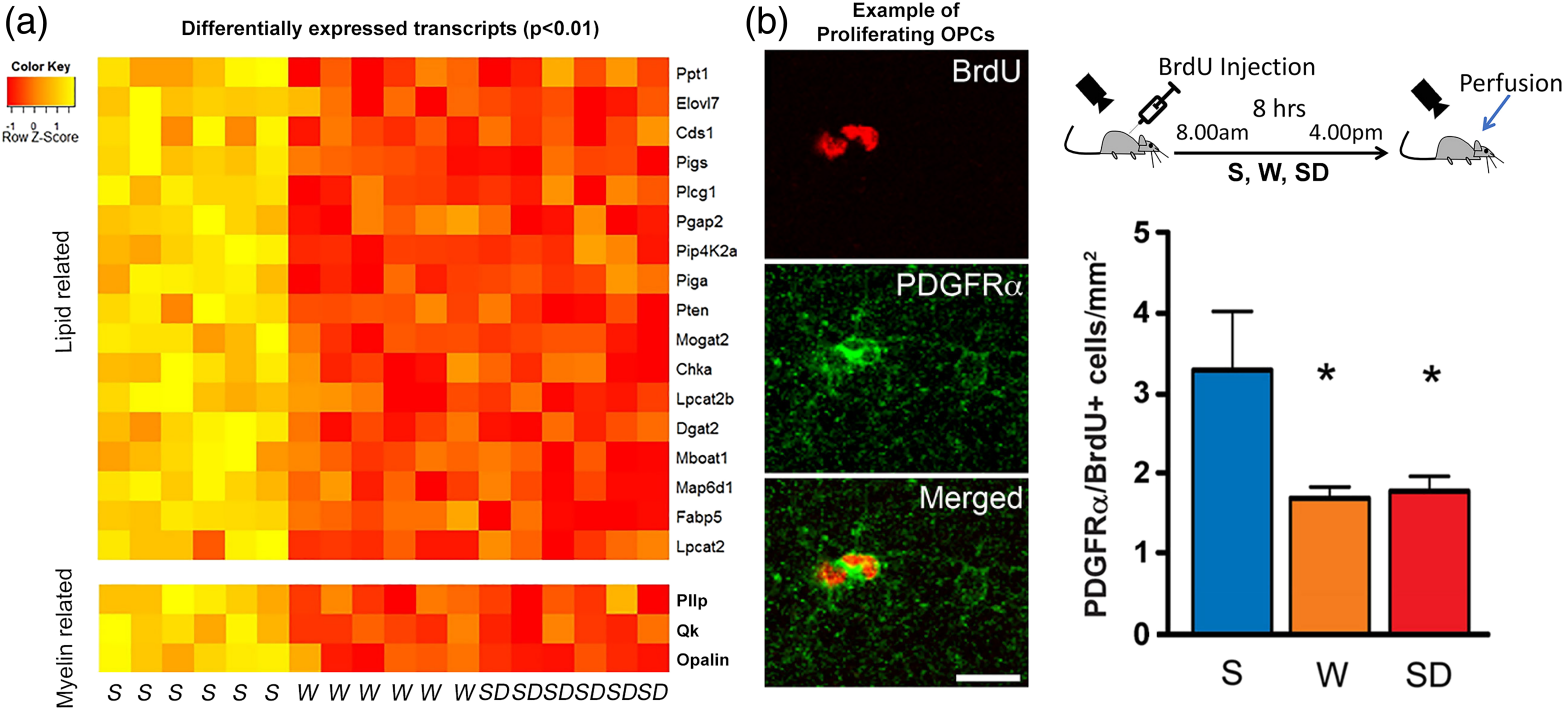
**Sleep and myelin**. (a). Heat diagram showing the expression intensity of differentially expressed transcripts of oligodendrocyte‐enriched forebrain samples of sleeping (S), awake (W), sleep deprived (SD). (b). Left, example of proliferating OPCs stained by the specific marker PDGFRα (green) and positive for BrdU (red). Scale bar: 15 μm. Right, scheme describing the experiment design (top) and quantification of proliferating OPCs in the cerebral cortex of mice after 8 hr of S, W, and SD (bottom). Adapted from Bellesi et al., 2013, 2015

By contrast, many genes implicated in apoptosis and cellular stress (*Acin1*, *Bcat1*, *Otud7b*, *Nr4a1*, *Hip1*, *Irf8*, *Traf6*, *HSPE1*, *Derl3*, *Hsp5*, *Golga3*, *Hsph1*, and *HSP90aa1*) were upregulated during spontaneous wake and short (~4 h) sleep deprivation relative to baseline sleep (Bellesi et al., 2013) (Figure 1). These data are in line with several experimental studies in humans and rodents showing that sleep loss exerts a strong regulatory influence on the immune system, promoting the secretion of a number of inflammatory mediators, such as IL‐1β and TNFα, which in turn can favor cellular stress, oxidative damage, and cytotoxicity (Hurtado‐Alvarado et al., 2013). Finally, a further functional cluster analysis more focused on OCP genes revealed that sleep was associated with the expression of transcripts (i.e., *Yap1*, *Mxd3*, and *Nrg2*) promoting OPC proliferation (Bellesi et al., 2013). Notably, Yap1 and Mxd3 interact with the Sonic hedgehog (Shh) signaling pathway, which is involved in OPC migration and proliferation (Barisone et al., 2008), and Nrg2, similarly to Nrg1, exerts its action by interacting with the ErbB family of receptors, whose activation is important for inducing OPC proliferation (Canoll et al., 1996). Thus, these results indicate that sleep favors myelination in different ways, ranging from new myelin deposition to lipid biosynthesis and turnover, and by promoting OPC proliferation.

### 2.2 Evidence from immunohistochemical and ultrastructural studies

The evidence that sleep can influence myelination is not limited to gene expression studies. In one experiment, we injected sleeping, spontaneously awake, and sleep deprived mice with Bromodeoxyuridine (BrdU), a compound that is incorporated in newly dividing cells and is commonly used to estimate cell proliferation (Bellesi et al., 2013). Subsequent analysis of BrdU+ cells that were also positive for the specific OPC marker PDGFRα (Platelet Derived Growth Factor Receptor Alpha) revealed that, in the cerebral cortex, the number of proliferating OPCs doubles during sleep relative to spontaneous wake and sleep deprivation (Figure 1). Conversely, the number of cells positive for O4, a marker expressed by premyelinating oligodendrocytes, was higher in wake than sleep, indicating that OPCs differentiation occurs primarily during wake (Bellesi et al., 2013). Therefore, the “wave” of OPCs proliferation that occurs during sleep (the day in mice) is followed by a “wave” of cell differentiation, which normally occurs during the major waking phase (the night in mice).

To understand which stage of sleep was responsible for promoting OPCs proliferation, BrdU quantifications were repeated in conjunction with electroencephalographic (EEG) recordings. These experiments revealed a positive correlation between the rate of OPCs proliferation and the amount of rapid‐eye‐movement (REM) sleep. No correlation was found with non‐REM sleep duration or non‐REM intensity, indicating that REM is the stage of sleep that favors OPCs proliferation (Bellesi et al., 2013). REM is abundantly present during early stages of development, when OPCs also profusely proliferate (El Waly et al., 2014). The mechanisms allowing REM sleep promotes OPC proliferation are unknown, but could be related to the action of the acetylcholine (Ach) (Bellesi, 2015), a neuromodulator whose extracellular levels are specifically high in REM sleep (MallIck/Inoue, 1999).

Ach regulates neurogenesis in the hippocampus via muscarinic receptor M1, M4, and α7, β2 nicotinic receptors (Bruel‐Jungerman et al., 2011). A recent study described a previously unknown population of choline acetyltransferase (ChAT+) neurons that innervate the subventricular zone (SVZ) and release ACh locally in an activity dependent manner. Optogenetic inhibition and stimulation of these cells in vivo modulated neurogenic proliferation in SVZ (Paez‐Gonzalez et al., 2014).

Similarly, in vitro studies demonstrated that ACh can induce OPC proliferation via the activation of ACh M1, M3, and M4 muscarinic receptors expressed on the cell bodies and processes of OPCs (De Angelis et al., 2012). However, during wake, a state in which the ACh tone is elevated as much as REM sleep, OPC proliferation seems to be inhibited. in vitro experiments indicated that the presence of noradrenaline (NA), the other major arousal neuromodulator, induces cytotoxicity and even apoptosis in OPCs through oxidative stress (Khorchid et al., 2002). Thus, the positive action of ACh on OPC proliferation may be blocked during wake because of the counteracting actions of NA.

Recently, we studied ultrastructural modifications of myelin in sleeping mice and in mice exposed to different regimes of sleep loss, from a few hours of sleep deprivation to ~5 days of chronic sleep restriction (Bellesi et al., 2018). Using electron microscopy, we analyzed more than 17,000 myelinated axons in two different brain regions—the corpus callosum (CC) and the lateral olfactory tract (LOT)—to obtain a comprehensive view of the effects of sleep loss on white matter. We found that short sleep deprivation does not lead to significant myelin changes; although on average axons appeared larger relative to sleep, this difference was not statistically significant. By contrast, when sleep deprivation was prolonged for 4.5 days with an absolute sleep reduction of about 70%, myelin thickness decreased by ~8%, while the diameter of axons remained comparable to baseline sleep. Interestingly, 32 hr of sleep recovery after the prolonged sleep deprivation were not sufficient to restore myelin thickness to baseline levels, indicating that the changes in myelin thickness associated with sleep and wake may require days to take place (Bellesi et al., 2018).

**Figure 2:**
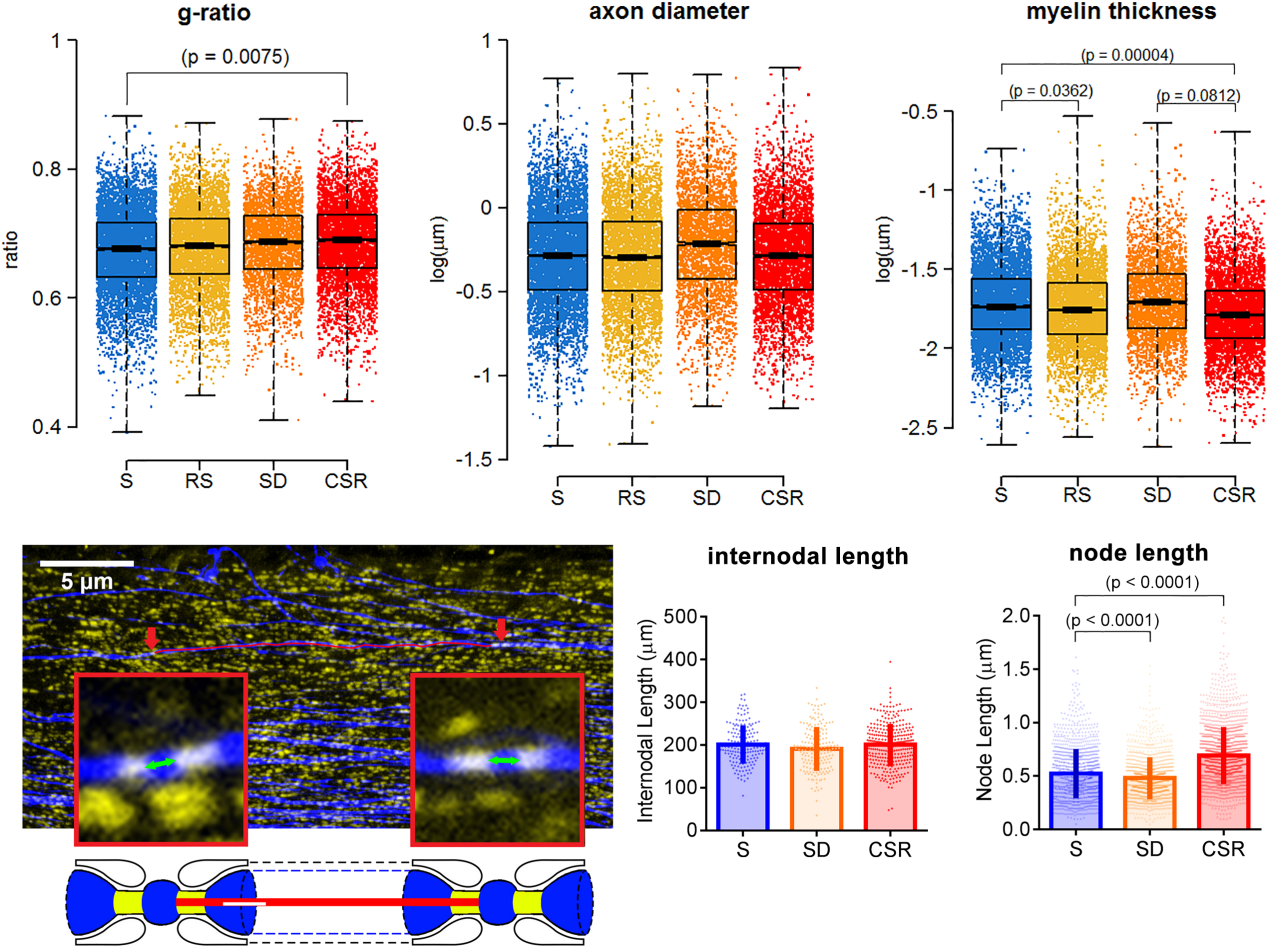
**Myelin changes after chronic sleep loss**. Top row: Distribution of g‐ratio, axon diameter, and myelin thickness measurements in adolescent mice after sleep (S), acute sleep deprivation (SD), chronic sleep restriction (CSR: ~5 days), recovery sleep (RS: 32 hr after the end of ~5 days of CSR). For axon diameter and myelin thickness, a log transformation has been applied to raw values, so the units are log(μm). Bottom row: Representative corpus callosum coronal section at the level of the decussation of the anterior commissure (+0.14 mm a/P from bregma) depicting YFP+ fibers (blue) and CASPR+ puncta (yellow). Red arrows indicate examples of nodes considered for the internodal length analysis, which are shown at larger magnification in the squared boxes. The red line along the axon indicates the internodal length, while the green arrows indicate node length. In the bottom scheme, representative nodes of Ranvier with paranodal sites (yellow) and the red line representing the internodal length are depicted. Graphs indicate distribution of internodal and node lengths values from the corpus callosum of S (*n* = 4 mice, 173 internodes and 1,412 nodes), SD (*n* = 4 mice, 180 internodes and 1,310 nodes), and CSR (*n* = 7 mice, 316 internodes and 2006 nodes) mice. Note that node length has been estimated as the shortest distance between two closely apposed CASPR+ puncta along the YFP+ axon. Data were analysed with ANOVA followed by Bonferroni's post hoc test. Adapted from Bellesi et al., 2018

In a different group of animals, we investigated the effects of short and long sleep deprivation on node and internodal length, two parameters that can impact conduction velocity. While the small changes in internodal length were not significant across the different groups (Bellesi et al., 2018), node length measurements showed a significant change in both short and prolonged sleep deprivation relative to sleep. Specifically, short sleep deprivation reduced node length by 8.4%, whereas prolonged sleep deprivation increased node length by ~32% (Figure 2). Computer simulations have showed that variations over a 4–8 fold range in node length can result in remarkable changes (up to 20%) in conduction velocity, similar to changes in myelin thickness or internode length (Arancibia‐Cárcamo et al., 2017). Since varying node length is by far a more energy‐efficient mechanism than modifying myelin thickness or internodal length to fine‐tune axon‐specific conduction velocity (Arancibia‐Cárcamo et al., 2017), it is possible that nerve cells actively alter the length of their nodes before variations in myelin thickness and internodal length can occur. Altogether, these data indicate that chronic lack of sleep widens the nodes of Ranvier and reduces myelin thickness. Both changes can reduce conduction velocity and might contribute to cognitive dysfunction associated with sleep loss.

Direct measurements of conduction velocity in ex‐vivo preparations of sleep deprived animals are lacking. However, event‐related potentials (ERPs), which are brain responses evoked by sensory stimuli typically used to assess information processing related to sensory, motor and/or cognitive functions (Ford and Pfefferbaum, 1991), are altered as a consequence of sleep loss. Two ERP components, N100 (also called N1) and P300 (also called P3) are large waves usually evoked with acoustic stimuli and reflecting the neuronal processing along the sensory pathways and within the primary and associative areas of the brain. The amplitude of both waves is reduced after sleep deprivation. As of P300, the ERP onset is also delayed after sleep deprivation (Morris et al., 1992; Lee et al., 2003). These findings, albeit indirectly, suggest that ERP modifications observed in conjunction with sleep loss could be accounted for, at least in part, by myelin changes.

**Figure 3:**
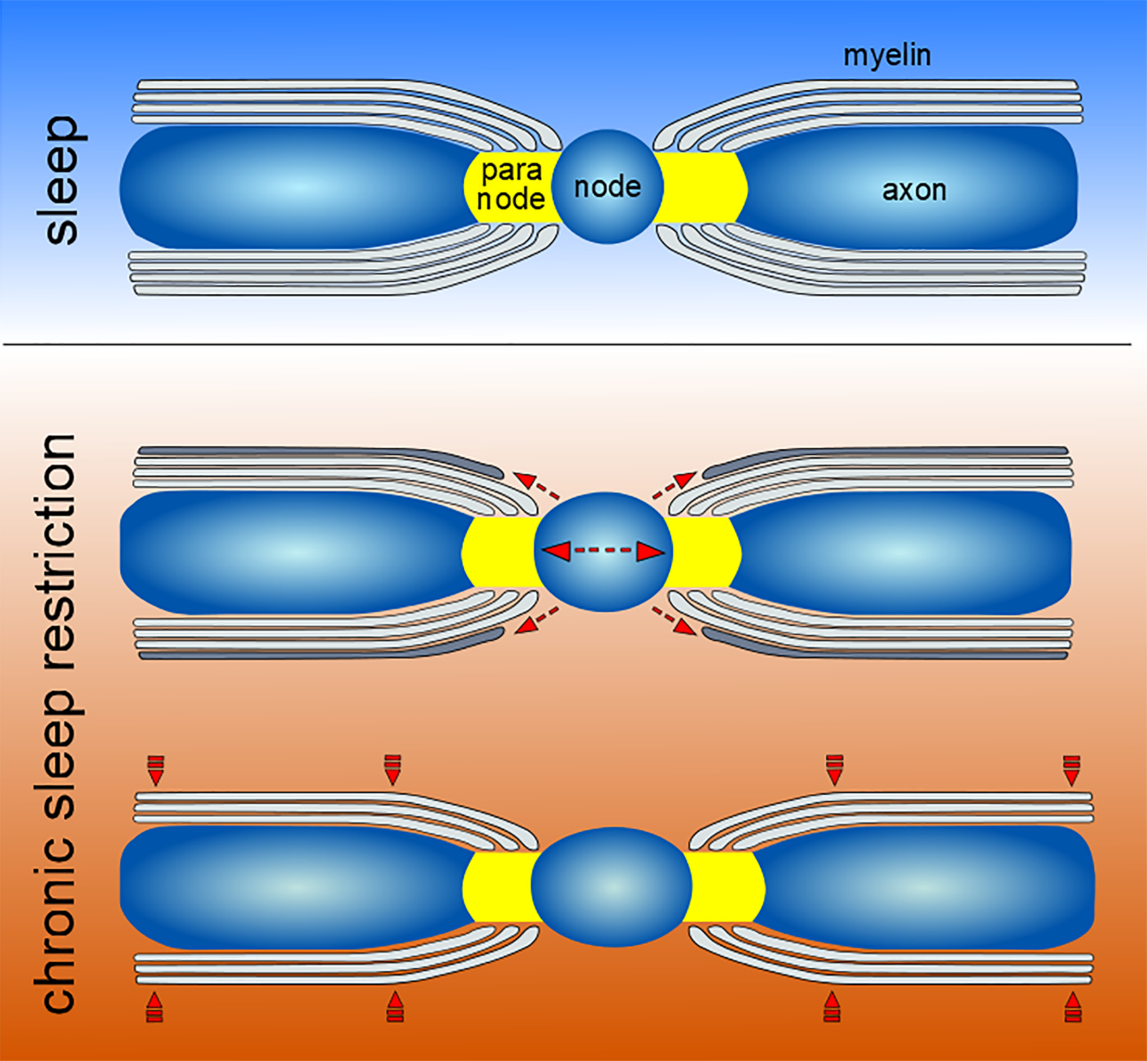
**How myelin is remodelled by chronic sleep loss**. A scheme representing the hypothetical mechanism underpinning node length widening and myelin thinning. As the chronic sleep restriction progresses, the outer paranodal loops (dashed thin arrows) first detach form the paranodal region, thus increasing the node length, and then retract into the oligodendrocyte with consequent myelin sheath thinning (dashed thick arrows)

Although the exact mechanism through which sleep loss affects myelin is unclear, a reasonable hypothesis is that the increase of the node length is caused by a detachment of the outer paranodal loop and retraction into the oligodendrocyte with subsequent myelin sheath thinning (see Figure 3). Recent evidence has shown that perinodal astrocytes can control the stability of the outer paranodal loop through release of the thrombin protease inhibitor PN1 (Serpine 2) that prevents thrombin‐dependent proteolysis of cell adhesion molecules (Neurofascin 155) that attach myelin to the axon (Dutta et al., 2018). Astrocytes are very sensitive to wake states, as demonstrated by the strong upregulation of many of their genes during wake, but also that their peripheral processes move and expand close to the synapses in response to wake periods of increasing duration (Bellesi et al., 2015a). While it is still uncertain whether perinodal astrocytic processes expand or retract with wake and sleep, a human genome‐wide association study (GWAS) involving 71,500 UK Biobank participants found a phenotypic association between reduced circadian rhythmicity, likely due to fragmented or disrupted sleep, and the genetic locus of Neurofascin, the cell adhesion molecule attaching the myelin to the axon (Ferguson et al., 2018).

### 2.3 Evidence from human studies

In humans, white matter changes are typically investigated using diffusion tensor imaging (DTI), an MRI‐based neuroimaging technique used to measure fractional anisotropy (FA). FA is a measure of water diffusivity in space and is based on the assumption that axonal and myelin membranes within fiber tracts constrain the normally random diffusion of water molecules to diffuse anisotropically along the lengths of the fibres (Beaulieu, 2002). Although changes in white matter FA can reflect myelin changes, additional factors such as axon diameter, fiber density, and organization can also contribute (Beaulieu, 2002). A wide range of human brain‐imaging studies in the past decade has clearly demonstrated that white matter structure can change after learning and functional experience (reviewed in Fields, 2015; Forbes and Gallo, 2017; Kaller et al., 2017; Monje, 2018). However, direct evidence on the role of sleep in promoting such structural modifications is limited.

Most of the studies focused on the link between white matter changes and sleep deprivation and quality. For example, in one study, young healthy subjects were DTI scanned in the morning after sleep and after 14 and 23 hr of wake. A widespread increase in FA was observed after 14 hr, whereas FA decreased after additional 9 hr of wake, with subjects displaying greater sleepiness showing also the largest decreases (Elvsåshagen et al., 2015). Another study examined whether differences in white matter microstructure may explain individual differences in vulnerability to sleep deprivation, finding that individuals more susceptible to sleep deprivation had lower FA values than less susceptible subjects in multiple brain regions (Rocklage et al., 2009). Similarly, higher FA values in the white matter of frontoparietal regions predicted better resistance to sleep deprivation (Cui et al., 2015). More recently, using a whole‐brain multimodal MRI approach 86 participants were investigated to explore the association between subjective sleep quality (PSQI global score) and functional and structural connectivity. Both measures decreased in association with poor sleep (Amorim et al., 2018). In related work, a structural MRI study carried out in 448 community‐dwelling older adults showed that poor sleep quality was associated with reduced global FA and increased global axial and radial diffusivity values of frontal white matter tracts (Sexton et al., 2017), thus suggesting that sleep quality is linked to white matter microstructure. However, the direction of any causal relationship between the two remains to be elucidated.

There is no doubt that sleep restriction can affect several aspects of waking cognitive performance with behavioral alertness being especially vulnerable (Banks and Dinges, 2007). Specifically, sleep loss is associated with an increased number of lapses and slower reaction times at a psychomotor vigilance task requiring sustained attention (Belenky et al., 2003; Van Dongen et al., 2003; Banks et al., 2010). In addition, the cumulative excess of wake induced by the chronic loss of sleep is near‐linearly related to the progressively reduced behavioral alertness on daytime performance, suggesting that the cognitive impairment due to sleep loss accumulates over days (Van Dongen et al., 2003). Working memory is also affected by reducing sleep. In one study, 14 days of chronic sleep restriction in healthy subjects significantly reduced the number of correct responses at digit symbol substitution task, a well‐established psychometric test paradigm, which is used to measure working memory performance. That impairment was proportional to the amount of sleep loss and length of sleep restriction, again indicating a cumulative effect of cognitive deficit over days (Van Dongen et al., 2003). These studies indicate that cognitive impairment associated with sleep loss is attributable to suboptimal functionality of frontal–parietal regions. Interestingly, these regions are also the ones showing more frequently white matter alterations in relation to insufficient sleep (Elvsåshagen et al., 2015; Sexton et al., 2017), thus raising the question whether the cognitive impairment induced by sleep loss could not depend, at least in part, on white matter modifications.

Additional evidence on the beneficial role of sleep for white matter comes from studies that investigated white matter in sleep disorders. For example, voxel wise between‐group comparisons of FA performed in 24 patients affected by primary insomnia and 35 healthy controls revealed decreased FA values within the right anterior internal capsule of the insomnia patients (Spiegelhalder et al., 2014). Likewise, a recent structural MRI study showed that white matter tracts of limbic and sensorimotor regions are disrupted in the right brain of patients with primary insomnia (Li et al., 2016), thus suggesting that right white matter tracts may be particularly sensitive to sleep disruption. Along the same line, there is ample evidence that obstructive sleep apnoea (OSA) leads to white matter alterations. A recent metanalysis systematically reviewed related research on the bidirectional relationship between OSA and white matter changes, showing that the patients with OSA had significantly more and more severe white matter changes, particularly in terms of white matter integrity, than those without OSA (Ho et al., 2018). In addition, histopathological analysis of 32 postmortem hippocampal samples of patients affected by OSA showed signs of cortical thinning and myelin loss. Interestingly, the patients that have used continuous positive airway pressure (CPAP) treatment showed no significant reductions in cortical thickness, but only myelin alterations, indicating that CPAP use may be protective against cortical thinning but not myelin loss (Kumar et al., 2014). It is worth noting that although sleep fragmentation associated with OSA can be severe and can negatively impact oligodendrocyte physiology and myelination, intermittent hypoxia likely plays a major role in this disease.

Although sleep may affect myelin, there is also evidence showing that primary deficits in myelin can lead to sleep and circadian disorders. For example, multiple sclerosis (MS), an autoimmune and degenerative disease characterized by CNS demyelination is frequently associated with sleep disorders, including insomnia, restless legs syndrome, periodic limb movement disorders, REM sleep behavior disorder, and sleep‐related breathing disorders (Veauthier, 2015; Foschi et al., 2019; Sakkas et al., 2019). Physical and psychological factors such as pain, anxiety and mood disorders, and nocturnal discomfort may all contribute to sleep disturbances in MS. However, the localization of demyelinating lesions may also play a role in determining the nature of the sleep disorder. For example, lesions at the brain stem and spinal cord level are more frequently associated with the appearance of REM sleep behavior disorder and restless legs syndrome (Foschi et al., 2019). Sleep fragmentation and alterations of circadian rhythms are common also in mice with experimental autoimmune encephalomyelitis, a mouse model of MS (Buenafe, 2012; He et al., 2014). Finally, a missense mutation in myelin oligodendrocyte glycoprotein (MOG) has been found in a rare familial form of narcolepsy (Hor et al., 2011), thus confirming that primary alterations in myelin may affect sleep.

### 2.4 Myelin vulnerability to sleep loss

Collectively, these findings indicate that myelin is continuously remodeled in the brain and sleep may be one of the key factors contributing to its formation and maintenance. On the other hand, these trophic effects on myelin could be compromised by the lack of adequate sleep. Although the mechanisms remain to be fully clarified, it is possible that sleep loss impairs myelin by altering specific oligodendrocyte functions. For example, it is plausible that oligodendrocytes prioritize some cellular functions at the expense of others (e.g., myelin maintenance and turnover) in conditions of high metabolic demand, like during periods of extended wake. Oligodendrocytes are extremely energetically demanding and are therefore vulnerable to energy deprivation (Rosko et al., 2018). For example, occlusion of the middle cerebral artery leads to oligodendrocyte swelling within 30 min and precedes neuronal necrosis by several hours (Pantoni et al., 1996). Accumulating evidence indicates that, besides forming and maintaining myelin, oligodendrocytes support axonal metabolism by shuttling energy‐related metabolites such as lactate to the axons (Philips and Rothstein, 2017). Thus, it is possible that fuelling neurons and supporting the integrity of myelinated axons during extended wake occur at the cost of myelin thinning. This would be in line with the fact that the transcript coding for plasmolipin, which helps assemble potential myelin membrane precursor domains at the Golgi complex (Yaffe et al., 2015) has been repeatedly found to be downregulated in sleep deprivation experiments (Cirelli et al., 2004; Mongrain et al., 2010; Bellesi et al., 2013).

The mechanisms through which sleep loss affects myelination might not occur entirely via energy budget reallocation. For example, it has been shown that even a few hours of sleep deprivation can induce the unfolded protein response, a cellular response consequent to the accumulation of un(mis)folded proteins at the endoplasmic reticulum (ER) (Naidoo et al., 2005). Such ER stress typically slows the synthesis of most proteins, thus creating an imbalance between increased need for supplies and impaired ability to produce them (Walter and Ron, 2011). Thus, it is also possible that prolonged wake limits new myelin deposition because of the induction of ER stress. Indeed, there is evidence that a large number of oligodendrocyte‐related genes (e.g., *Derl3*, *Hsp5*, *Hsph1*), which are upregulated by sleep deprivation are involved in the degradation of misfolded proteins and, more in general, participate in the process of the unfolded protein response (Oda et al., 2006; Naidoo et al., 2007; Meares et al., 2008; Wang et al., 2009).

Another possibility is that the effects of sleep loss on myelin are mediated by the behavioral stress inherently associated with sleep loss. In this regard, several myelin‐related transcripts have been found to be downregulated by psychosocial stress, a chronic behavioral stress that in mice is usually modeled by the chronic social defeat stress paradigm (Laine et al., 2018). Of note, these stress‐related transcriptional changes were consistently associated with modifications in myelin thickness in some specific regions (e.g., medial prefrontal cortex, ventral hippocampus, etc.) (Laine et al., 2018). In other studies, different types of chronic stress such as repeated water immersion and restraint stress led to structural modifications of the oligodendrocytes in mice. These morphological changes were not associated with significant changes in myelin thickness, but with a reduction of the node and paranodal lengths (Yamamoto et al., 2016). Furthermore, four weeks of chronic variable stress in mice increased the transcription of myelin‐related genes (e.g., *Mag*, *Mobp*) in the corpus callosum, whereas it reduced the expression of other myelin genes (*Mog* and *Mbp*) in medial prefrontal cortex and nucleus accumbens, thus suggesting a complex and region dependent response of oligodendrocytes to behavioral stress (Liu et al., 2017). Notably, no sleep evaluation has been performed before, during, and after the induction of stress in none of these studies. Therefore, it is difficult to know whether the myelin‐related effects are then primarily due to behavioral stress or to the coexisting sleep disruption that can be induced by the stressing procedures (Pawlyk et al., 2008; Olini et al., 2017).

Finally, sleep loss could affect oligodendrocytes and myelin through the involvement of other glial cells. For example, astrocytes are known to support oligodendrocyte functions and myelination in several ways (Kıray et al., 2016). Astrocytes synthesize lipids that can be shuttled to and used by oligodendrocytes to produce myelin (Pfrieger and Ungerer, 2011). Indeed, mice lacking the ability to synthetize lipids in astrocytes display a persistent hypomyelination across neurodevelopment (Camargo et al., 2017; Ferris et al., 2017). Moreover, astrocytes can modulate OPC proliferation and differentiation by providing soluble factors such as platelet derived growth factor (PDGF) and fibroblast growth factor 2 (FGF‐2), or by releasing a variety of cytokines (e.g., tumor necrosis factor alpha [TNFα]) and chemokines (e.g., C‐X‐C motif chemokine 10 [CXCL10]) (Kıray et al., 2016). All these astrocytic functions are likely modulated by sleep loss, although direct evidence is still lacking. In any case, there are good reasons to believe that sleep loss strongly influences astrocytes. For instance, a recent study showed that the extracellular space in cortex shrinks considerably during wake, limiting metabolite clearance and suggesting changes in astrocytic morphology (Xie et al., 2013; O'Donnell et al., 2015). Other studies found that astrocytes are activated by prolonged wake. Genes related to metabolism and lactate shuttling, extracellular matrix, cytoskeleton, and phagocytosis are upregulated after a few hours of sleep deprivation (Petit et al., 2010; Bellesi et al., 2015a, b). In addition, sleep loss prompts the astrocytes to structurally remodel synapses and unmyelinated axons by phagocyting portions of these structures (Bellesi et al., 2017). Although astrocytes were found to possess lipid inclusions which contained myelin in the human optic nerve (Nag and Wadhwa, 2012), it is not clear whether their increased phagocytic activity induced by sleep loss also results in myelin phagocytosis and remodeling. Microglial cells are also activated by sleep loss (Hsu et al., 2003; Bellesi et al., 2017). Activated microglia can release cytokines such as interleukin (IL)‐1β, IL‐6, and TNF‐α and elevated levels of these proinflmmatory cytokines have been found associated with sleep deprivation in both rodents and humans (Mullington et al., 2010; Kincheski et al., 2017; Manchanda et al., 2018). IL‐1β and TNF‐α negatively modulate OPC proliferation and survival and ultimately may limit de‐novo myelination (Selmaj and Raine, 1988; Vela et al., 2002; Schmitz and Chew, 2008). In addition, cytokines released by microglia can modulate astrocytes, which in turn influence OPC responses (Moore et al., 2015). Finally, activated microglia represents a major source of reactive oxygen species (Colton et al., 2000), which may contribute to oxidative stress in OPCs and oligodendrocytes, thus limiting myelination.

In conclusions, oligodendrocytes may express a particular vulnerability to sleep loss, which may depend on their elevated metabolism and high susceptibility to incur oxidative stress and/or may be mediated by other glial cells. By contrast, sleep may represent a favorable moment for oligodendrocytes to reestablish cellular homeostasis and promote myelin growth. Consequently, sleep could play an important role in the process that leads to increased myelination following learning or increased neuronal activation. In this case, myelin remodeling takes place typically over multiple days (Gibson et al., 2014; McKenzie et al., 2014). It is possible therefore that new myelin deposition occurs preferably during sleep when oligodendrocytes are less likely engaged in supporting sustained axonal firing and subjected to cellular stress.

## 3 CONCLUSIONS

Transcriptomic studies in rodents have revealed that many transcripts coding for proteins involved in myelination, lipid metabolism, and OPC proliferation are upregulated in the sleeping brain, indicating an overall beneficial function of sleep for myelin maintenance and turnover. These data have been corroborated by additional studies that evaluated the consequences of restricted sleep on myelin and white matter in general. We have reviewed evidence in both rodents and humans that appears to confirm that the loss of sleep is detrimental for myelin, thus supporting the hypothesis that one of the functions of sleep may be indeed to maintain healthy white matter. However, mechanisms through which sleep acts to favor myelination have not yet been systematically mapped. Moreover, while it is well established that sleep plays fundamental roles in learning and memory, it is less clear whether sleep is a necessary requirement for promoting adaptative myelination following learning. Further research addressing these important questions will be critical in advancing our understanding of how sleep regulates brain functions and why sleep is indispensable for our well‐being.

## CONFLICT OF INTEREST

The authors have no conflict of interest to declare.
